# Clearance of Heparan Sulfate and Attenuation of CNS Pathology by Intracerebroventricular BMN 250 in Sanfilippo Type B Mice

**DOI:** 10.1016/j.omtm.2017.05.009

**Published:** 2017-06-06

**Authors:** Mika Aoyagi-Scharber, Danielle Crippen-Harmon, Roger Lawrence, Jon Vincelette, Gouri Yogalingam, Heather Prill, Bryan K. Yip, Brian Baridon, Catherine Vitelli, Amanda Lee, Olivia Gorostiza, Evan G. Adintori, Wesley C. Minto, Jeremy L. Van Vleet, Bridget Yates, Sara Rigney, Terri M. Christianson, Pascale M.N. Tiger, Melanie J. Lo, John Holtzinger, Paul A. Fitzpatrick, Jonathan H. LeBowitz, Sherry Bullens, Brett E. Crawford, Stuart Bunting

**Affiliations:** 1 Research, BioMarin Pharmaceutical Inc., 105 Digital Drive, Novato, CA 94949, USA

**Keywords:** lysosomal storage disorder, mucopolysaccharidosis IIIB, MPS IIIB, Sanfilippo B, enzyme replacement therapy, intracerebroventricular delivery, glycosaminoglycan, neurodegeneration, mental retardation

## Abstract

Sanfilippo syndrome type B (mucopolysaccharidosis IIIB), caused by inherited deficiency of α-*N*-acetylglucosaminidase (NAGLU), required for lysosomal degradation of heparan sulfate (HS), is a pediatric neurodegenerative disorder with no approved treatment. Intracerebroventricular (ICV) delivery of a modified recombinant NAGLU, consisting of human NAGLU fused with insulin-like growth factor 2 (IGF2) for enhanced lysosomal targeting, was previously shown to result in marked enzyme uptake and clearance of HS storage in the *Naglu*^*−/−*^ mouse brain. To further evaluate regional, cell type-specific, and dose-dependent biodistribution of NAGLU-IGF2 (BMN 250) and its effects on biochemical and histological pathology, *Naglu*^*−/−*^ mice were treated with 1–100 μg ICV doses (four times over 2 weeks). 1 day after the last dose, BMN 250 (100 μg doses) resulted in above-normal NAGLU activity levels, broad biodistribution, and uptake in all cell types, with NAGLU predominantly localized to neurons in the *Naglu*^*−/−*^ mouse brain. This led to complete clearance of disease-specific HS and reduction of secondary lysosomal defects and neuropathology across various brain regions lasting for at least 28 days after the last dose. The substantial brain uptake of NAGLU attainable by this highest ICV dosage was required for nearly complete attenuation of disease-driven storage accumulations and neuropathology throughout the *Naglu*^*−/−*^ mouse brain.

## Introduction

Sanfilippo syndrome type B (Sanfilippo type B, mucopolysaccharidosis IIIB, or MPS IIIB) is a rare inherited lysosomal storage disorder caused by mutations in the gene encoding α-*N*-acetylglucosaminidase (NAGLU, EC 3.2.1.50), an enzyme required for the degradation of the glycosaminoglycan heparan sulfate (HS).^1^ NAGLU deficiency leads to lysosomal accumulation of partially degraded HS, primarily in cells of the CNS.^2^ Clinically, the disease is predominantly characterized by progressive, severe cognitive and neurological deterioration with relatively less profound somatic manifestations compared with other types of MPS disease. Patients with Sanfilippo type B usually succumb to the disease during the second and third decades of life.^3^, ^4^ To date, no disease-modifying treatments have been approved. However, several investigational therapies are currently being developed that aim to clear the pathological accumulation of HS, thereby slowing or preventing the subsequent progression of clinical symptoms.^5^, ^6^, ^7^

Restoration of functional NAGLU activity in the CNS, the primary site of Sanfilippo type B pathology, requires delivery of a therapeutic gene or protein across the blood-brain barrier (BBB), a major challenge that must be overcome. Potential CNS-directed gene therapy approaches have recently shown promising preclinical results in Sanfilippo type B animal models.^8^, ^9^, ^10^, ^11^, ^12^, ^13^ The safety, tolerability, and translatability of therapeutic potential to human patients, however, are currently being evaluated (http://www.isrctn.com, ISRCTN #19853672).^14^ Brain-directed enzyme replacement therapy, by BBB-penetrating technology^15^, ^16^ or cerebrospinal fluid (CSF)-directed administration, is an alternative potential approach to restore NAGLU function. CSF delivery of therapeutic proteins, such as intracerebroventricular (ICV) injection, has recently been reported to be tolerated and effective in clinical^17^ and preclinical^18^, ^19^ studies of neuropathic lysosomal storage diseases, including Sanfilippo type B.^20^

When infused directly into a cerebral ventricle of Sanfilippo type B (*Naglu*^*−/−*^) mice, a modified form of NAGLU (NAGLU-IGF2), comprising human NAGLU recombinantly fused to insulin-like growth factor 2 (IGF2) for IGF2/mannose 6-phosphate (M6P) receptor-mediated enhanced lysosomal targeting,^21^, ^22^, ^23^ results in marked enzyme uptake in the brain.^20^ ICV administration of NAGLU-IGF2 cleared the pathologic storage of partially degraded HS in the brain with no sign of re-accumulation for at least 1 month after the last dose and reduced secondary accumulations of previously identified pathologic markers, such as β-hexosaminidase and lysosome-associated membrane protein 1 (LAMP1).^20^, ^24^, ^25^, ^26^, ^27^ Notably, complete normalization of pathologic HS storage in the brain was achievable despite the fact that the enzyme was mainly taken up by neurons^23^ and significantly less taken up into microglia, where large storage vacuoles were previously identified.^26^

In the present study, we further evaluated the ability of ICV-delivered NAGLU-IGF2 (BMN 250) to access different regions and cell types within the *Naglu*^*−/−*^mouse brain to ameliorate not only the primary HS storage but also secondary pathological changes, including lysosomal defects, reactive astrocytosis, and neuroinflammation. In addition, we assessed the dose responses to delineate the critical relationship between the extent of global treatment effects within the brain as a whole and pathological correction in specific regions of the brain.

## Results

### The IGF2 Tag Mediates Uptake of BMN 250 into Cultured Mouse Neurons

**Figure 1:**
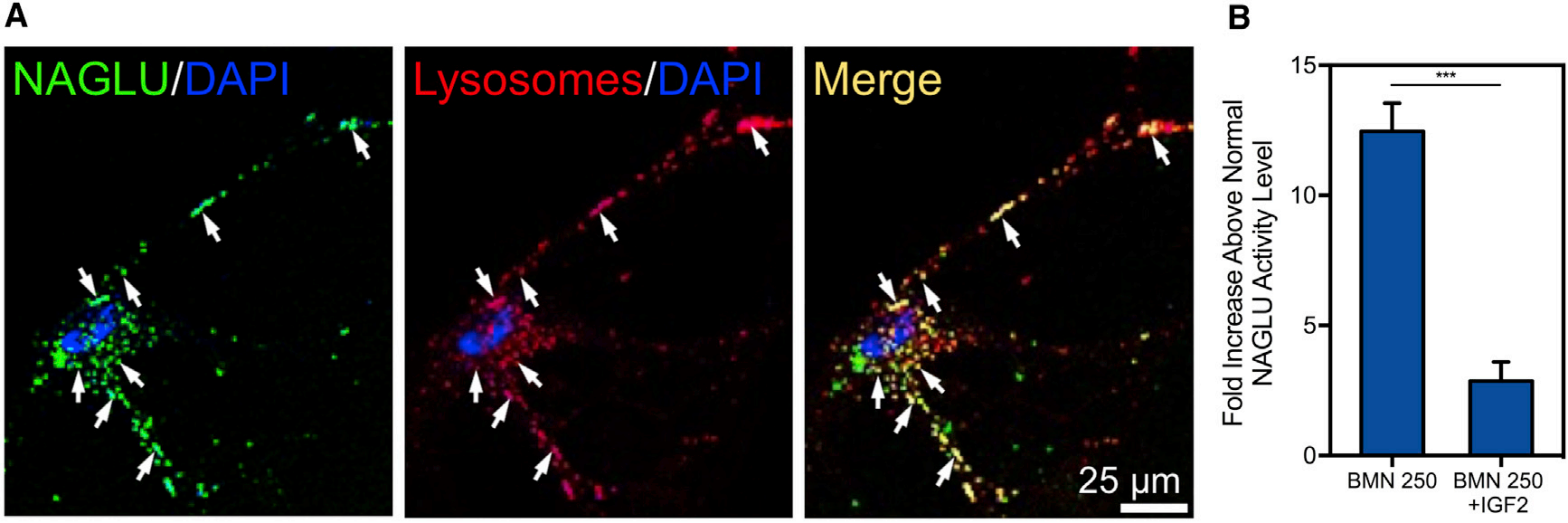
In Vitro Uptake of BMN 250 in Normal Mouse Hippocampal Neurons (A) Representative confocal images of cultured mouse hippocampal neurons treated with Alexa Fluor 488-labeled BMN 250 (green) and LysoTracker red (red), showing localization of NAGLU signals to lysosomes (yellow), with arrows indicating representative co-localized signals. (B) IGF2/mannose 6-phosphate receptor-dependent uptake of BMN 250 by normal mouse neurons was significantly inhibited by IGF2. NAGLU activity in cell lysates, expressed as the fold elevation (mean ± SD, n = 3) above normal levels. ***p < 0.001.

BMN 250 was readily endocytosed by normal mouse hippocampal neurons in vitro, leading to high levels of enzyme activity in the cells (Figures 1A and 1B). Internalized BMN 250 was partially co-localized with LysoTracker dye, used for lysosomal labeling (Figure 1A). The uptake of BMN 250 was extensively inhibited (77.0% ± 5.9%; mean ± SD) by excess IGF2, indicating that the neuronal cell uptake and lysosomal targeting occurred via IGF2/M6P receptor-mediated endocytosis (Figure 1B).

### ICV-Delivered BMN 250 Is Broadly Distributed and Predominantly Taken up by Neurons

**Figure 2:**
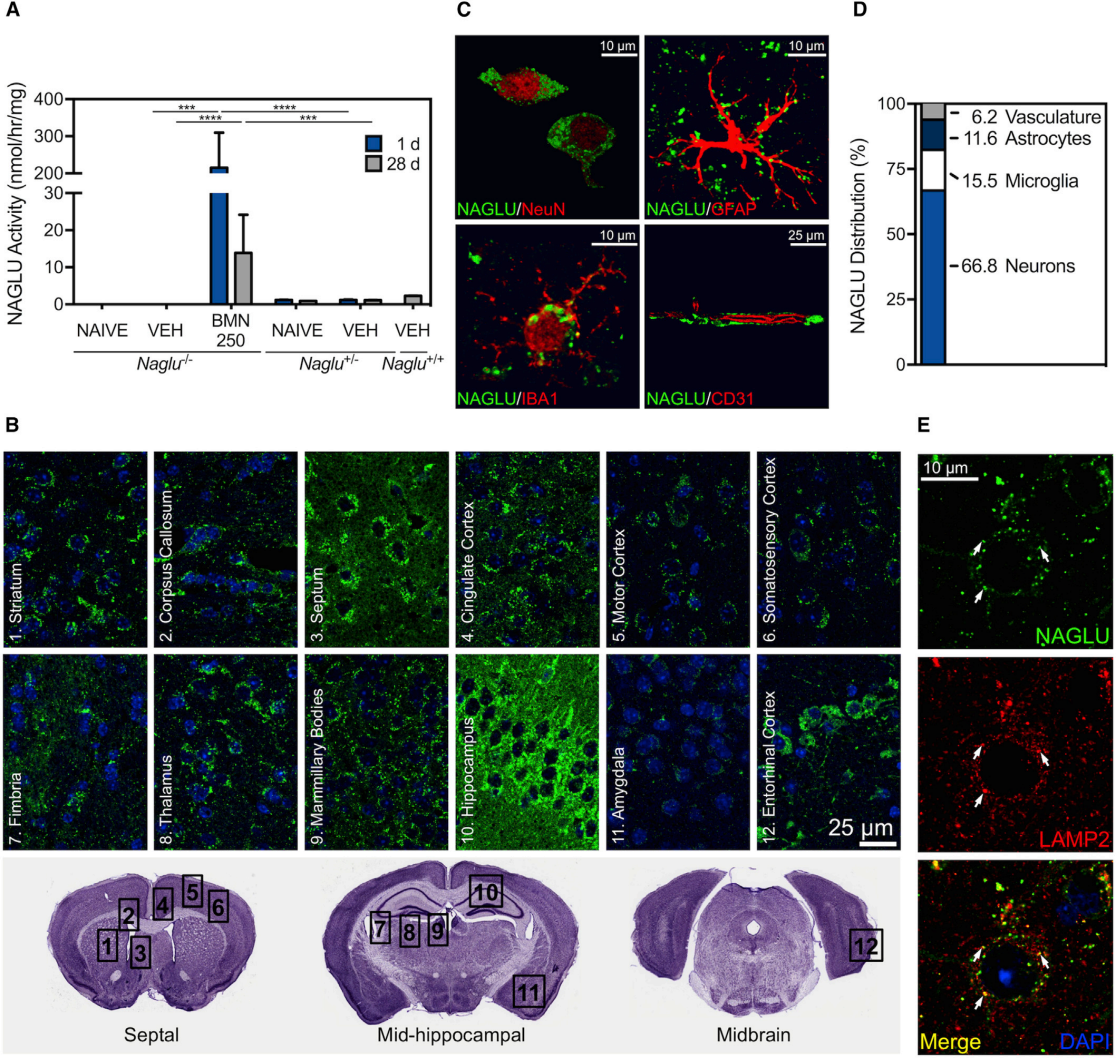
Uptake and Biodistribution of BMN 250 in *Naglu*^*−/−*^ Mouse Brain *Naglu*^*−/−*^ mice were administered 100 μg ICV doses of BMN 250 or vehicle (controls) on study days 1, 5, 10, and 14. n = 6–8 animals per treatment group, except n = 2 for the naive *Naglu*^*+/−*^ group. (A) NAGLU enzyme activity (mean ± SEM) measured in brain homogenate 1 or 28 days after the last ICV infusion. VEH, vehicle. ***p < 0.001, ****p < 0.0001. (B) Immunohistochemical signal of NAGLU (green) with DAPI nuclear stain (blue) in the septal, mid-hippocampal, and midbrain sections, showing bilateral biodistribution across the neuro-axis 1 day after the last ICV dose. (C) Representative high-resolution confocal images of NAGLU (green) co-stained with NeuN, GFAP, IBA1, and CD31 (red), showing localization of the NAGLU signal to neurons, astrocytes, microglia, and endothelia, respectively, 1 day after the last ICV infusion. (D) Percent of total NAGLU signal associated with each cell type, determined by quantitative analysis on co-stained sections 1 day after the last ICV infusion. (E) Representative high-resolution confocal images of NAGLU (green) co-immunostained with LAMP2 (red), showing localization of the NAGLU signal to lysosomes (yellow), indicated by arrows, 1 day after the last ICV infusion.

Repeated administration of BMN 250 (four 100 μg doses given over a 2-week period) to the lateral ventricle of *Naglu*^*−/−*^ mice resulted in markedly elevated levels of NAGLU enzyme activity in homogenized brain tissue. The increased activity in the BMN 250-treated *Naglu*^*−/−*^ mouse brain, up to 200-fold above that of the unaffected *Naglu*^*+/+*^ controls 24 hr after the final ICV infusion, remained 6- to 12-fold above the normal levels 28 days post-infusion (Figure 2A). When brain slices from these treated mice were qualitatively evaluated by immunohistochemistry, broad biodistribution of BMN 250 was evident in all animals 1 day following the last infusion (Figure 2B). Although inter-animal variability was noted (n = 6–8), as in the case of the measured levels of enzyme activity (Figure 2A), NAGLU immunostaining was generally distributed bilaterally and broadly across the neuro-axis in regions including the septum, striatum, corpus collosum, cortex (cingulate, motor, somatosensory, and entorhinal), hippocampus, fimbria, mammillary bodies, thalamus, and amygdala (Figure 2B).

Cell type and subcellular distribution of ICV-administered BMN 250 (four 100 μg doses over a 2-week period) in *Naglu*^*−/−*^ mouse brains 24 hr after the last dose was evaluated by double-staining immunohistochemistry. Co-immunostaining of NAGLU with specific markers of key cell types in the CNS indicates that BMN 250 is taken up by neurons (neuronal nuclei [NeuN]), astrocytes (glial fibrillary acidic protein [GFAP]), microglia (ionized calcium-binding adaptor molecule 1 [IBA1]) and endothelia (CD31) (Figure 2C). Based on quantitative assessment, BMN 250 appears to be predominantly localized to neurons (Figure 2D). In the cingulate cortex, for example, 66.8% of the total NAGLU signal was found to be associated with neurons, leaving the remaining 15.5%, 11.6%, and 6.2% to microglia, astrocytes, and endothelia, respectively (Figure 2D). Furthermore, the immunostaining for NAGLU was co-localized with a lysosomal protein called LAMP2 (Figure 2E), indicating lysosomal delivery of BMN 250. Taken together, ICV infusions of BMN 250 to *Naglu*^*−/−*^ mice result in widespread brain uptake of enzymatically active NAGLU in all key cell types, with most of NAGLU distributing to neurons.

### ICV-Delivered BMN 250 Results in Complete Clearance of Pathological Heparan Sulfate and Normalization of Lysosomal Storage Pathology

**Figure 3:**
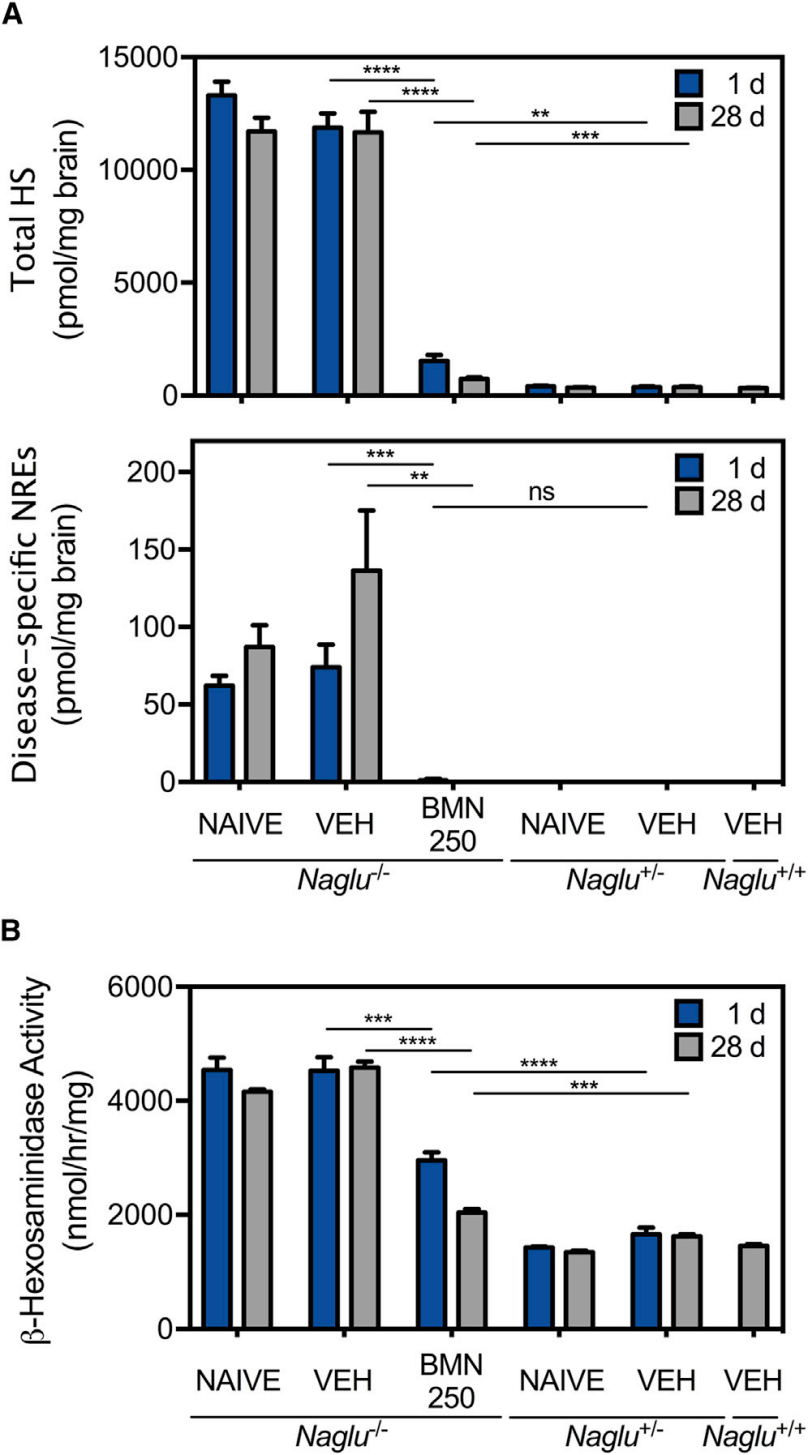
Reduction of Lysosomal Storage and Elevated β-hexosaminidase Activity by BMN 250 at 1 and 28 Days following Four 100 μg ICV Doses over 2 Weeks (A) Steady-state levels for total HS and disease-specific NREs in brain homogenate, shown as picomoles per milligram of tissue wet weight (mean ± SEM). (B) β-Hexosaminidase enzyme activity (mean ± SEM) measured in brain homogenate. VEH, vehicle. n = 6 per group, except n = 2 for naive *Naglu*^+/−^ mice. **p < 0.01, ***p < 0.001, ****p < 0.0001; ns, not significant.

At 16 weeks of age, *Naglu*^*−/−*^ mouse brains (untreated or vehicle-treated) showed highly elevated steady-state levels of total HS and disease-specific HS-chain non-reducing ends (NREs) with the trisaccharide A0I2S0^28^ (*N*-acetyl glucosamine 2-sulfated iduronic acid *N*-sulfated glucosamine) compared with normal levels in *Naglu*^*+/−*^ and *Naglu*^*+/+*^ controls (untreated or vehicle-treated) (Figure 3A). The HS NREs are derived specifically from HS fragments that have been partially degraded in the lysosome, generating a terminal *N*-acetyl-glucosamine residue that cannot be degraded further because of the deficiency of NAGLU.^28^ Intraventricular treatments of *Naglu*^*−/−*^ mice with BMN 250 (four 100 μg doses given over a 2-week period) resulted in a precipitous drop of 87.1% ± 2.2% and 98.5% ± 1.4% (mean ± SEM) in total HS and NREs, respectively, relative to the levels of vehicle-treated *Naglu*^*−/−*^ mice 24 hr after the last infusion (Figure 3A). This reduction persisted for at least 28 days, during which total HS levels were only 2-fold higher than those of unaffected controls, and NRE levels were no longer detectable (Figure 3A).

BMN 250 treatments also led to a notable reduction of β-hexosaminidase activity, which is typically increased in *Naglu*^*−/−*^ mouse tissues.^25^ β-Hexosaminidase enzyme activity, elevated at least 2-fold above normal in *Naglu*^*−/−*^ mouse brains, significantly decreased over time for 28 days after the treatments (four 100 μg doses given over a 2-week period) to levels closer to (1.4- to 1.6-fold) those in *Naglu*^*+/−*^ and *Naglu*^*+/+*^ control mouse brains (Figure 3B).

**Figure 4:**
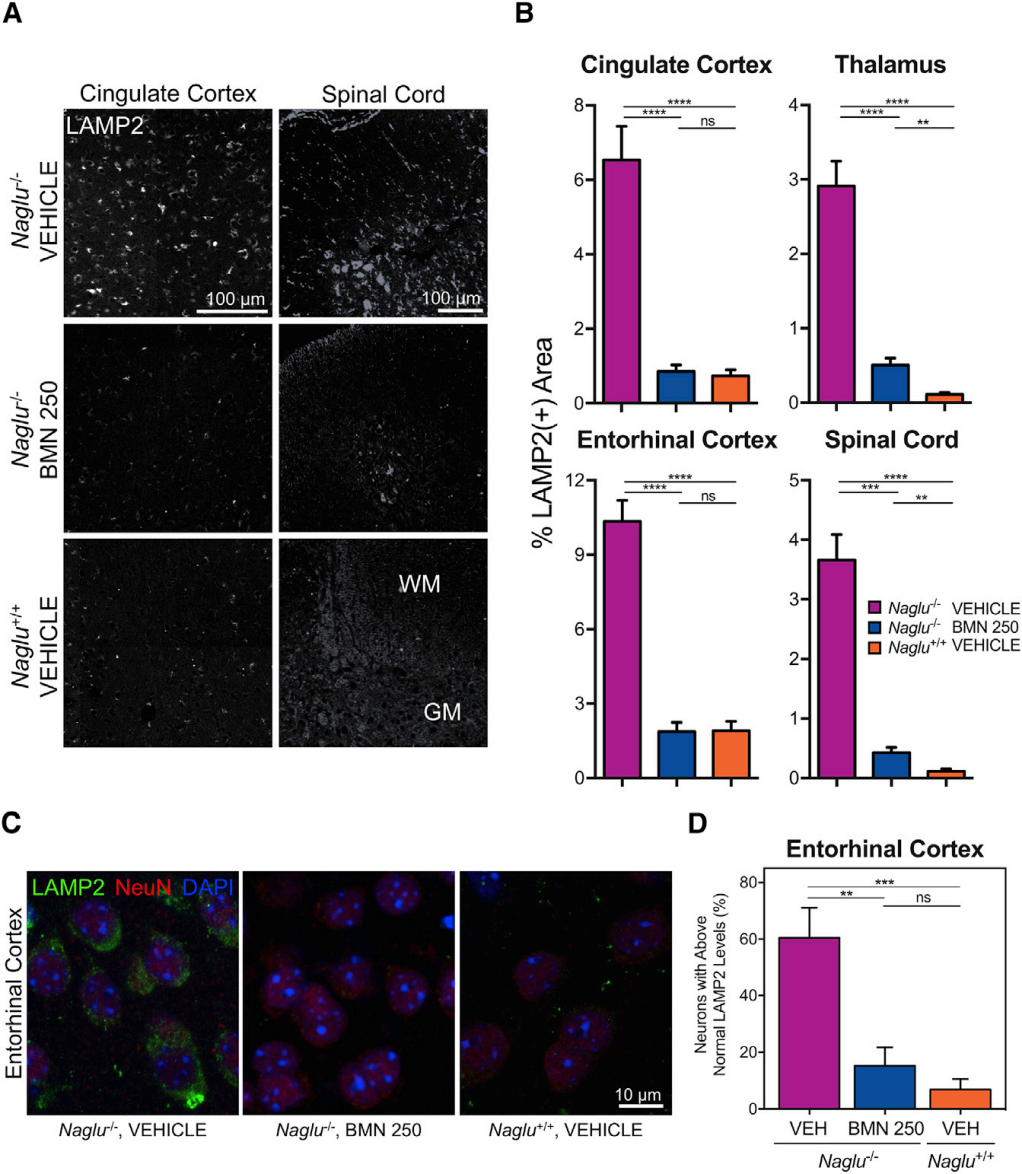
Reversal of Lysosomal Storage Pathology by BMN 250 in Different Brain Regions and Spinal Cord Harvested at 28 Days following Four 100 μg ICV Doses over 2 Weeks (A) Representative images of immunostaining for LAMP2 (white) in the cingulate cortex and spinal cord (WM, white matter; GM, gray matter). (B) Quantitative analyses of LAMP2 signals (mean ± SEM, n = 5–8 animals per group) in the cingulate cortex, thalamus, entorhinal cortex, and spinal cord. (C) Representative high-resolution confocal images of immunostains for LAMP2 (green), NeuN (red), and DAPI (blue) in the entorhinal cortex for qualitative evaluation of neuronal LAMP2 signals. (D) Percentage of entorhinal cortex neurons (NeuN-positive) with LAMP2 signal above the normal (mean of wild-type values ± 1 SD) level (mean ± SEM, n = 8 animals per group). VEH, vehicle. **p < 0.01, ***p < 0.001, ****p < 0.0001.

Reduction of lysosomal storage pathology was also demonstrated by immunohistochemical changes of LAMP2 (Figure 4A), a commonly used marker to evaluate alteration of lysosome number and size. Quantitative analyses of the cingulate cortex, thalamus and entorhinal cortex indicated that the percent total area positive for LAMP2 was significantly increased in vehicle-treated *Naglu*^*−/−*^ mice (6.54% ± 0.90%, 2.91% ± 0.33%, and 10.34% ± 0.85%, respectively; mean ± SEM) compared with the values measured for the wild-type controls (0.74% ± 0.17%, 0.11% ± 0.03%, and 1.90% ± 0.38%, respectively; mean ± SEM) (Figure 4B). Treatments with BMN 250 (four 100 μg doses given over a 2-week period) reduced these elevated LAMP2 signals in the cingulate cortex, thalamus, and entorhinal cortex by 88% on average (0.86% ± 0.17%, 0.51% ± 0.09%, and 1.87% ± 0.37%, respectively; mean ± SEM), close to normal levels 28 days after the last infusion (Figure 4B). The normalization effects of BMN 250, evident across the different brain regions, were also apparent in the spinal cord (Figure 4A), distal to the site of ICV treatment. The percent total LAMP2-positive area in the spinal cord sections from enzyme-treated *Naglu*^*−/−*^ mice was significantly reduced by >90% (0.43% ± 0.08%, mean ± SEM) relative to levels in vehicle-treated *Naglu*^*−/−*^ mice (3.66% ± 0.43%, mean ± SEM), closer to the value of the wild-type controls (0.12% ± 0.04%, mean ± SEM) (Figure 4B).

Effects of ICV-delivered BMN 250 on LAMP2 levels of the entorhinal cortex were further examined in neurons, to which it is preferentially targeted^20^ (Figure 2D). The entorhinal cortex was previously reported as a specific brain area with elevation of markers of neurodegeneration in *Naglu*^*−/−*^ mice.^26^ Co-immunostaining with LAMP2 and NeuN demonstrated that the entorhinal cortical neurons of vehicle-treated *Naglu*^*−/−*^ mice showed increased levels of LAMP2 (Figures 4C and 4D). This neuron-specific LAMP2 signal was completely normalized in *Naglu*^*−/−*^ mice 28 days after BMN 250 treatment (four 100-μg ICV infusions in a 2-week period) (Figures 4C and 4D). When quantified as a percentage of the entorhinal cortical neurons that contained higher LAMP2-positive levels than the normal level measured in *Naglu*^*+/+*^ mice, the accumulations of neuronal LAMP2 in the *Naglu*^*−/−*^ mice were effectively reduced from 60% ± 11% to 15% ± 7% (mean ± SEM) by ICV-administered BMN 250 (Figure 4D).

### ICV-Delivered BMN 250 Reduces Neuroinflammation and Reactive Astrocytosis

**Figure 5:**
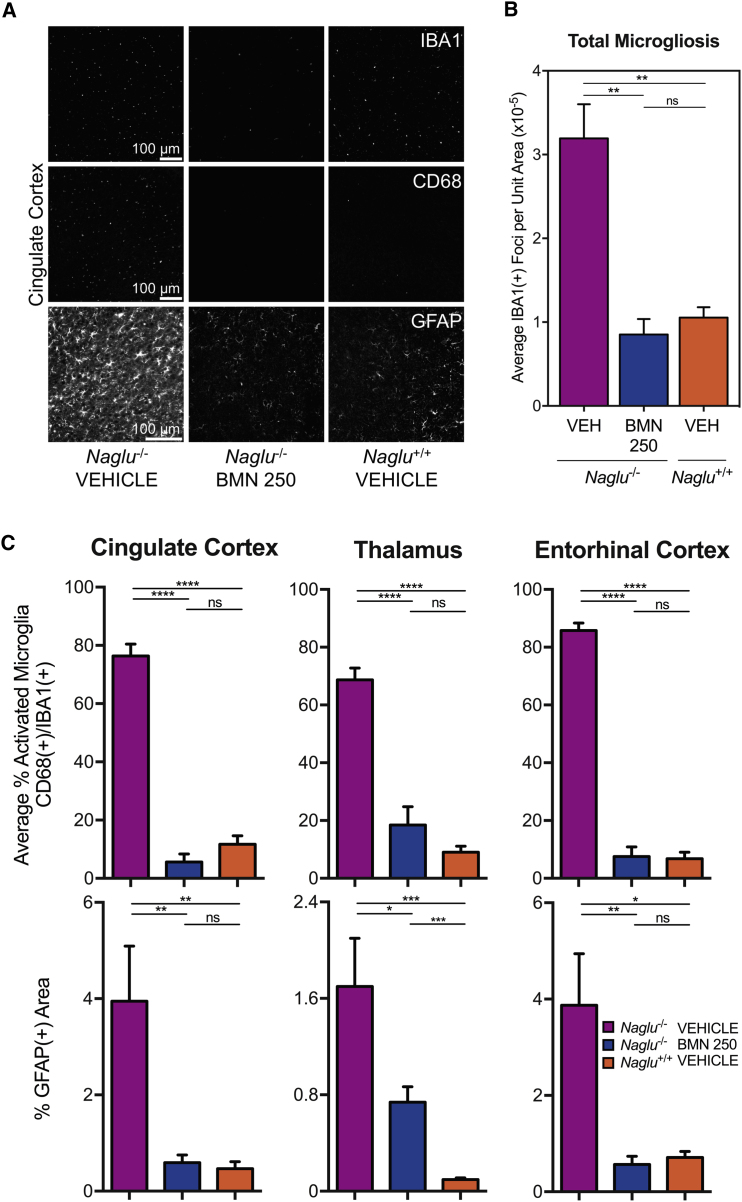
Effects of BMN 250 on Neuro-inflammation and Reactive Astrocytosis at 28 Days following Four 100 μg ICV Doses over 2 Weeks (A) Representative images from the cingulate cortex of immunohistochemical signals (white) for IBA1, CD68, and GFAP. (B) Quantification of total microglia^29^ (IBA1-positive foci). (C) Quantification of actively phagocytic microglia^30^, ^31^ (percent CD68-positive/IBA1-positive) and astrocytosis (percent GFAP-positive area) in the cingulate cortex, thalamus, and entorhinal cortex (mean ± SEM). VEH, vehicle. n = 6–8 animals per treatment group. *p < 0.05, **p < 0.01, ***p < 0.001, ****p < 0.0001.

In addition to the correction of primary pathologic storage of HS and associated lysosomal defects, ICV infusion of BMN 250 (100 μg dose) repeated four times over a 2-week period effectively reversed the neuroinflammation typically observed in the *Naglu*^*−/−*^ mouse brain^26^ (Figure 5A). Brain sections of vehicle-treated *Naglu*^*−/−*^ mice at euthanasia (28 days after the final ICV infusion) showed that total microglia,^29^ measured as the total number of IBA1-positive foci per unit area, was substantially increased by ∼300% (3.19 ± 0.41, mean ± SEM) relative to levels observed in *Naglu*^*+/+*^ controls (1.06 ± 0.12, mean ± SEM) (Figure 5B). 28 days following the last BMN 250 infusion, the elevation in total microglia was completely normalized (0.85 ± 0.18, mean ± SEM) to the control level (Figures 5A and 5B). Subsequent co-immunohistochemistry assessment demonstrated that BMN 250 also alleviated microglial activation, determined as the percentage of IBA1-positive cells (total microglia)^29^ that were also positive for CD68 (actively phagocytic microglia)^30^, ^31^ (Figures 5A and 5C). All three brain regions (cingulate cortex, thalamus, and entorhinal cortex) examined from the vehicle-treated *Naglu*^*−/−*^ mice exhibited considerably more activated microglia (76.39% ± 4.06%, 68.69% ± 4.11%, and 85.75% ± 2.62%, respectively; mean ± SEM), compared with those observed in unaffected *Naglu*^*+/+*^ mice (11.70% ± 2.88%, 9.07% ± 2.09%, and 6.77% ± 2.29%, respectively; mean ± SEM) (Figure 5C). 28 days following ICV treatment with 100 μg doses of BMN 250 given four times over a 2-week period, microglia activation was no longer observed in the cingulate cortex, thalamus, and entorhinal cortex of *Naglu*^*−/−*^ mice (5.64% ± 2.71%, 18.49% ± 6.27%, and 7.57% ± 3.30%, respectively; mean ± SEM) (Figure 5C).

BMN 250 also improved reactive astrocytosis (Figures 5A and 5C). Astrocytosis, measured as the increase in the total area positive for GFAP immunostaining, is evident in multiple brain regions (cingulate cortex, thalamus, and entorhinal cortex) evaluated for vehicle-treated *Naglu*^*−/−*^ mice (3.95% ± 1.14%, 1.70% ± 0.40%, and 3.87% ± 1.07%, respectively; mean ± SEM) compared with those of unaffected normal mice (0.47% ± 0.14%, 0.10% ± 0.01%, and 0.71% ± 0.13%, respectively; mean ± SEM) (Figure 5C), as previously reported.^32^ 28 days after the four ICV infusions with 100 μg doses given over two weeks, significant reductions of ∼70% on average in GFAP immunoreactivity, relative to the vehicle-treated *Naglu*^*−/−*^ group, were observed in the cingulate cortex, thalamus, and entorhinal cortex of BMN 250-treated *Naglu*^*−/−*^ mice (0.59% ± 0.16%, 0.74% ± 0.13%, and 0.57% ± 0.17%, respectively; mean ± SEM) (Figure 5C).

### ICV-Delivered BMN 250 Exhibits Dose-Dependent Correction in Lysosomal Storage Pathology

**Figure 6:**
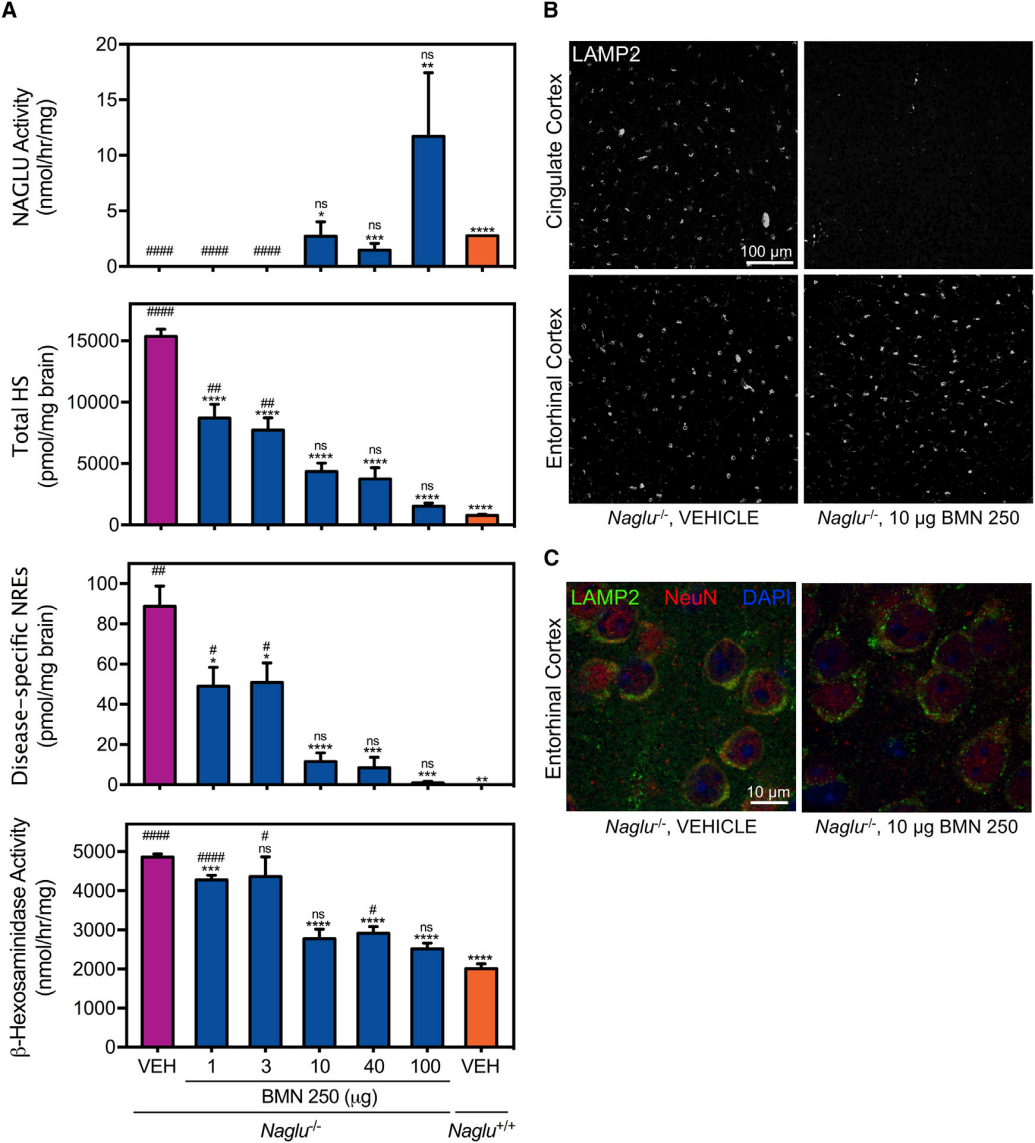
Dose Response of BMN 250 on Lysosomal Storage Pathology at 28 Days following 1, 3, 10, 40, or 100 μg ICV Doses Four Times over 2 Weeks (A) NAGLU catalytic activity, steady-state total HS and disease-specific NRE biomarkers, and β-hexosaminidase enzyme activity measured in brain homogenate (mean ± SEM; VEH, vehicle-treated *Naglu*^*−/−*^ mice; n = 11; 1 and 3 μg BMN 250, n = 6; 10 μg BMN 250, n = 10; 40 and 100 μg BMN 250, n = 4; VEH *Naglu*^*+/+*^, n = 2. Bottom: *p < 0.05, **p < 0.01, ***p < 0.001, ****p < 0.0001 versus VEH *Naglu*^*−/−*^. Top: ^#^p < 0.05, ^##^p < 0.01, ^####^p < 0.0001 versus VEH *Naglu*^*+/+*^. (B) Representative images of LAMP2 immunostain (white) from the cingulate cortex and entorhinal cortex, harvested from *Naglu*^*−/−*^ mice treated with 10 μg doses of BMN 250 or vehicle (controls). (C) Representative high-resolution confocal images of immunostains for LAMP2 (green), NeuN (red), and DAPI (blue) in the entorhinal cortex of *Naglu*^*−/−*^ mice treated with 10 μg doses of BMN 250 or vehicle (controls) for qualitative evaluation of neuronal LAMP2 signals.

When *Naglu*^*−/−*^ mice were treated with different ICV doses (1, 3, 10, 40, or 100 μg) four times in a 14-day period, the extent of uptake of BMN 250 into brain tissues generally depended on the ICV infusion dosage. At doses above 10 μg, BMN 250 resulted in restoration of NAGLU enzyme activity in the *Naglu*^*−/−*^ mouse brain that stayed at or above the vehicle-treated normal *Naglu*^*+/+*^ control levels for 28 days following the last treatment (Figure 6A). Significant differences in NAGLU activity were not observed among dosing groups ≥ 10 μg because of inter-animal variability within each group (p = 0.13, 100 μg versus 40 μg; p = 0.16, 100 μg versus 10 μg; p = 0.56, 40 μg versus 10 μg).

The effects of enzyme replacement on biochemical pathology demonstrated distinct dose-response relationships. Increasing the dose of BMN 250 (1, 3, 10, 40, or 100 μg) progressively decreased the steady-state levels of both the total HS and disease-specific NREs in the *Naglu*^*−/−*^ mouse brains 28 days following four ICV infusions in 2 weeks (Figure 6A). With the lowest doses below 10 μg, moderate reductions of approximately 50% and 30% in the total HS and NRE levels, respectively, were achieved compared with vehicle-treated *Naglu*^*−/−*^ mice. At the 10-μg dose, greater reductions of 70% and 90% in total HS and NRE levels, respectively, were observed relative to the vehicle-treated *Naglu*^*−/−*^ mice. Finally, at the highest dosage (100 μg), the total HS and NRE signals were indistinguishable from those of vehicle-treated unaffected *Naglu*^*+/+*^ controls (Figure 6A).

Interestingly, the higher ICV doses that led to notable total HS and NRE reductions were required to decrease elevated levels of β-hexosaminidase activity in the *Naglu*^−/−^ mouse brain. In *Naglu*^−/−^ mice treated with ≥10 μg of BMN 250, β-hexosaminidase activity in the brain was only 1.3- to 1.5-fold above the normal level of the *Naglu*^+/+^ controls, a significant improvement from the 2.4-fold elevated activity measured in vehicle-treated *Naglu*^−/−^ mice (Figure 6A), 28 days following the four ICV treatments in a 2 week-period.

### Clearance of Lysosomal Storage throughout the CNS Is Attainable Only by the Highest ICV Doses of BMN 250

Regional dose-response effects of ICV-delivered BMN 250 were evaluated by analyzing changes in immunohistochemical signals of LAMP2 in different brain areas (cingulate cortex and entorhinal cortex) 28 days following four ICV treatments given in a 14-day period. In the cingulate cortex near the enzyme administration site, the 10 μg dosing level, corresponding to an apparent threshold dose for notable decreases in accumulated HS and elevated β-hexosaminidase activity in brain homogenate (Figure 6A), was sufficient for a marked reduction in the number of LAMP2-positive foci relative to the vehicle-treated *Naglu*^−/−^ mouse brain (Figure 6B). Surprisingly, in the entorhinal cortex, relatively distal to the ICV infusion site, the same 10 μg doses resulted in no qualitative difference in the amount of LAMP2-positive signal from mice treated with vehicle 28 days after the last dose (Figure 6B). This lack of reduction of the LAMP2 signal in the entorhinal cortex was evident even in neurons (Figure 6C), the major cell type to which BMN 250 is distributed predominantly^20^ (Figure 2D). The observed regional variability of normalization effects by the 10 μg doses was strikingly different from the higher 100 μg doses, which decreased the elevated LAMP2 signal in all brain regions and the spinal cord (Figure 4). Collectively, the substantial brain uptake of NAGLU via ICV delivery of high-dose BMN 250 (e.g., 100 μg) leads to complete clearance of the primary storage of HS in brain tissue; when this is achieved, secondary pathological accumulations appear to be normalized across widespread areas of the brain.

## Discussion

We and others have previously reported that CSF-directed delivery of recombinant lysosomal enzyme resulted in marked enzyme uptake in the brain of mouse disease models, where it functioned to degrade the primary lysosomal storage.^20^, ^33^, ^34^, ^35^, ^36^ In the present study, we further evaluated regional, cell type-specific, and dose-dependent distribution of ICV-delivered recombinant human NAGLU-IGF2 fusion protein and its effectiveness in normalizing primary lysosomal storage and secondary brain pathology.

The data presented here confirm our earlier observations that the NAGLU signal is indeed predominantly localized in neurons (Figure 2D), which presumably only represent 20%–50% of all cells in the brain.^37^, ^38^ The disproportionately high level of neuronal uptake suggests that BMN 250 undergoes selective targeting, likely mediated by binding of its IGF2 moiety to the receptor^39^ (Figure 1B) known to be widely expressed in neurons,^40^ as discussed previously.^20^ Our present data also confirm that the remaining NAGLU signal is distributed among other cell types of the brain, such as microglia, astrocytes, and vasculature. Identification of the NAGLU signal in all key cell types, along with broad bilateral biodistribution (Figure 2B), is consistent with our repeated observations that the ICV treatments can effectively lead to normalization of accumulated HS in the *Naglu*^*−/−*^ mouse brain^20^ (Figure 3A).

The present study demonstrates that the significant and long-lasting reduction of the stored HS in the adult mouse brain by short-course repeated ICV administration^20^ can still be attained at lower doses of BMN 250. Doses of 10 μg (∼0.4 mg/kg body weight) given repeatedly over 2 weeks resulted in reductions of ∼65% and ∼80% of the accumulated HS and NREs, respectively, in the brain relative to vehicle-treated affected mice (Figure 6A), accompanied by improvement in neuropathology in brain regions, such as cingulate cortex (Figure 6B). Moreover, these effects lasted for at least 28 days without additional treatment, hence generally supporting both BMN 250 and the ICV administration route as promising therapeutic developments.

The superiority of the highest dose of BMN 250 (100 μg or ∼4 mg/kg body weight) was evident by its remarkable normalization effects across multiple brain regions on the elevated LAMP2 signal, a commonly used, indirect marker of lysosomal storage in *Naglu*^*−/−*^ mice.^26^, ^32^ The most noticeable dose-dependent response was observed in the entorhinal cortex (Figures 4B–4D and 6B, and 6C), the specific brain region of *Naglu*^*−/−*^ mice previously reported to show a number of pathological defects involving neurons, including upregulation of markers of neurodegeneration^24^, ^41^ and prominent accumulation of LAMP2 signal in neuronal soma.^42^ We previously observed that the neuronal LAMP2 signal in the entorhinal cortex was already increased in 2-week-old *Naglu*^*−/−*^ mice, suggesting that this may be an early marker of neuronal damage and dysfunction.^42^ Our present data therefore indicate that lower ICV doses, which can apparently lead to notable global reduction of lysosomal storage (Figure 6A), may still not be sufficient to ameliorate the regional lysosomal storage pathology in the critical areas of the brain (Figures 6B and 6C).

In conclusion, based on dose-dependent, regional normalization of lysosomal storage as a marker of residual disease, disease correction in Sanfilippo type B is more likely attained when nearly complete reduction of primary pathologic storage of HS is achieved by ICV delivery of substantial levels of NAGLU throughout the CNS. These compelling preclinical findings warrant clinical evaluation of the safety, tolerability, and therapeutic potential of BMN 250 as a potential ICV enzyme replacement therapy for Sanfilippo type B (https://www.clinicaltrials.gov: NCT 02754076 and 02493998).

## Materials and Methods

### Enzyme

Recombinant human NAGLU-IGF2 fusion protein was expressed in Chinese hamster ovary cells and purified as described elsewhere^20^ (US 9,376,480 B2). The purified protein (20 mg/mL) was stored frozen at −80°C in artificial cerebrospinal fluid solution (vehicle): 1 mM Na_2_HPO_4_/NaH_2_PO_4_, 148 mM NaCl, 3 mM KCl, 0.8 mM MgCl_2_, and 1.4 mM CaCl_2_ (pH 7.2). Prior to ICV infusion, the enzyme was diluted, where necessary, with the vehicle solution to final concentrations (0.2–20 mg/mL) to maintain the equivalent dosing volume throughout the studies.

### Enzyme Activity Assays

NAGLU enzyme activity was determined using a synthetic fluorogenic substrate, 4-methylumbelliferyl (4MU)-*N*-acetyl-α-glucosaminide (EMD Millipore or Toronto Research Chemicals), following a published protocol^43^ with minor modifications.^20^ β-Hexosaminidase activity was similarly determined using 4MU-*N*-acetyl-β-glucosaminide (EMD Millipore), as described previously.^20^ Activity was expressed as nanomoles of 4MU released per hour per total protein, which was estimated by Bradford assay with BSA as standard.

### In Vitro Cell Uptake Analyses

Normal mouse-derived embryonic day 17 hippocampal neurons (Lonza) were incubated with 100 nM BMN 250 in the absence or presence of 4 μM IGF2 (Cell Sciences) for 24 hr. NAGLU activity in cell lysates was assayed using the 4MU substrate as described above and expressed as the fold increase above normal levels. For co-localization experiments, neurons were incubated with 100 nM BMN 250, conjugated with Alexa Fluor 488 (Thermo Fisher Scientific) for 24 hr. Cells were then incubated with 200 nM LysoTracker red (Thermo Fisher Scientific), following the manufacturer’s protocols, and then fixed with paraformaldehyde and analyzed by confocal imaging.

### Animals and BMN 250 Administration

All mouse study protocols were approved by the Institutional Animal Care and Use Committee. Transgenic mice lacking the enzyme NAGLU^25^ (*Naglu*^*−/−*^, B6.129S6-*Naglu*^*tm1Efn*^/J, The Jackson Laboratory, 00827), heterozygous littermates (*Naglu*^+/−^), and wild-type controls (*Naglu*^*+/+*^*, C57BL/6J*, The Jackson Laboratory, 00664) were enrolled in this study to receive either BMN 250 or vehicle twice weekly for 2 weeks via ICV infusion. All mice were approximately 16 weeks of age at study start. Groups were randomized with respect to gender and body weight.

Methods for ICV administration have been described previously.^20^ Briefly, 5 days prior to study start, a permanent cannula was surgically placed into the left lateral ventricle of the brain of each mouse. ICV infusions (5 μL total volume over a period of 15 min) were administered via the implanted cannula on study days 1, 5, 10, and 14 to deliver either BMN 250 doses from 1–100 μg or vehicle. Mice were euthanized either 1 or 28 days following the final infusion. Brain samples were collected and homogenized in water for biochemical analysis. For histological analysis, in situ fixation with 10% paraformaldehyde was performed prior to tissue collection.

### Quantification of Total and Disease-Specific Heparan Sulfate

Total and Sanfilippo type B-specific HS NREs in homogenized brain samples were quantified using the Sensi-Pro assay, following previously published procedures.^44^, ^45^ Briefly, brain HS glycosaminoglycans were purified by anion exchange chromatography and digested with heparan lyases (IBEX Technologies). The depolymerization products were then tagged with isotopically labeled aniline by reductive amination and quantified by liquid chromatography-mass spectrometry. The measured quantity of total HS (internal disaccharides) and disease-specific NREs (trisaccharides, *N*-acetyl glucosamine 2-sulfated iduronic acid *N*-sulfated glucosamine, referred to as A0I2S0^28^) was expressed as picomoles per milligram of brain wet weight.

### Immunohistopathological Analyses

Formalin-fixed, paraffin-embedded coronal brain sections from approximately bregma 0.75 mm (septal), bregma −1.75 mm (mid-hippocampal), and bregma −4.60 mm (midbrain) and thoracic and lumbar spinal cord cross-sections were immunostained using a Ventana Discovery Ultra autostainer (Ventana Medical Systems). Antibodies used in the study included rabbit anti-NAGLU (0.4 μg/mL, BioMarin Pharmaceutical), mouse anti-NeuN (1:100, EMD Millipore), mouse anti-GFAP (1:500, Sigma-Aldrich), rabbit anti-GFAP (1:5,000, Sigma-Aldrich), goat anti-IBA1 (1:1600, Abcam), goat anti-CD31 (1:200, R&D Systems), rat anti-LAMP2 (1:100, Abcam), and rabbit anti-CD68 (1:200, Abcam). Representative confocal images were acquired on a Leica TCS SP8 confocal microscope with an HC PL APO 40×/1.30 or 63×/1.4 oil objective and 1-AU pinhole diameter (Leica Microsystems). Unless otherwise noted, quantitative image analysis was performed using ImageJ.^46^ All statistics were analyzed in GraphPad Prism 6 (GraphPad).

Regional and cell type-specific distribution of BMN 250 in the coronal brain sections was evaluated by immuno-staining with anti-NAGLU alone or plus antibodies to cell type-specific markers: NeuN, GFAP, IBA1, or CD31. Whole sections were scanned using the Leica Ariol slide scanning microscope with a HC PL APO 20×/0.7 objective (Leica Microsystems), and regions for image analysis were extracted bilaterally from two rostral-caudal extents of the cingulate cortex at approximately bregma 0 mm and bregma −2 mm. The percent distribution of NAGLU signal in astrocytic, microglial, or endothelial compartments was derived from the NAGLU signals contained within GFAP-, IBA1-, or CD31-positive signal relative to the total signal. Custom ImageJ^46^ macros were used to quantify the overlap. The neuronal NAGLU signal was calculated by subtracting the sum of the NAGLU signal contained in the astrocytic, microglial, and endothelial compartments from the total NAGLU signal. Lysosomal localization of the NAGLU signal was evaluated by co-immunostaining with NAGLU and LAMP2 antibodies.

Lysosomal pathology, reactive astrocytosis, and neuroinflammation were analyzed by immuno-staining the coronal brain sections with LAMP2, GFAP, IBA1, and CD68 antibodies. Regions from the whole scanned sections were extracted bilaterally from the cingulate cortex, thalamus, and entorhinal cortex for image analysis. For the spinal cord analyses, the entire cross-sections from thoracic and lumbar regions were evaluated. LAMP2 signals localized in neuronal compartments of the midbrain-level coronal section were analyzed by co-immunostaining for NeuN and LAMP2; confocal z stacks were acquired in the dorsal and ventral entorhinal cortex of all animals. Volocity software (PerkinElmer) was used to quantify the volume of LAMP2 associated with NeuN-positive neurons. Normal neuronal LAMP2 levels were determined by averaging the LAMP2 volume within neurons of control mice and adding 1 SD.

## Author Contributions

M.A.-S., D.C.-H., R.L., J.V., G.Y., T.M.C., P.A.F., J.H.L., S. Bullens, B.E.C., and S. Bunting designed research; D.C.-H., J.V., G.Y., H.P., B.K.Y., B.B., C.V., A.L., O.G., E.G.A., W.C.M., J.L.V.V., B.Y., S.R., P.M.N.T., M.J.L., and J.H. performed research; M.A.-S., D.C.-H., R.L., J.V., G.Y., H.P., B.K.Y., C.V., E.G.A., J.L.V.V., B.Y., S.R., T.M.C., P.M.N.T., M.J.L., J.H., and S. Bullens analyzed data; M.A.-S., D.C.-H., R.L., J.V., G.Y., S. Bullens, B.E.C., and S. Bunting prepared the manuscript.

## Conflicts of Interest

All authors are employees of and have equity interest in BioMarin Pharmaceutical Inc., which is developing BMN 250 as a potential commercial therapeutic agent. M.A.-S., T.M.C., and J.H.L., together with others, hold a patent for compositions and methods for treating Sanfilippo type B, comprising therapeutic NAGLU-IGF2 fusion proteins (US 9,376,480 B2).
